# Comparative lesion metrics analysis of very high power and high power short duration radiofrequency ablation in a Porcine ex vivo model

**DOI:** 10.1038/s41598-025-06533-5

**Published:** 2025-06-20

**Authors:** Yannick Teumer, Henrik Ziemssen, Lyuboslav Katov, Carlo Bothner, Benjamin Mayer, Wolfgang Rottbauer, Karolina Weinmann-Emhardt

**Affiliations:** 1 Ulm University Heart Center, Albert-Einstein-Allee 23, Ulm, Germany; 2 Institute for Epidemiology and Medical Biometry, Ulm University, Schwabstraße 13, Ulm, Germany

**Keywords:** Cardiac electrophysiology, Ablation, Radiofrequency, Lesion metrics, Very-high-power, High-power, Cardiology, Interventional cardiology

## Abstract

High-power, short-duration (HP-SD) ablation is a well-established radiofrequency (RF) ablation protocol in cardiac electrophysiology. Recently, very high-power, short-duration (vHP-SD) ablation has emerged as an alternative. This study compares lesion metrics between vHP-SD and HP-SD ablation protocols using the latest irrigated RF catheter with temperature-based power regulation, considering the impact of contact force (CF). RF ablations were performed in a porcine ex vivo model using myocardial preparations in a circulating saline bath. Three protocols were applied: vHP-SD (90 W for 4s), HP-SD-4 (50 W for 4s) and HP-SD-15 (50 W for 15s). A total of 360 lesions in 12 hearts were analyzed (vHP-SD: 120; HP-SD-4: 120, HP-SD-15: 120). The HP-SD-4 protocol produced the lowest mean lesion depth (2.42 ± 0.61 mm vs. 3.16 ± 0.41 mm vs. 4.49 ± 0.66 mm, *p* < 0.001), mean maximum lesion diameter (6.02 ± 1.00 mm vs. 7.34 ± 0.92 mm vs. 9.13 ± 1.59 mm), and mean lesion volume (52.0 ± 22.5 mm³ vs. 96.4 ± 28.8 mm³ vs. 211.4 ± 95.3 mm³, *p* < 0.001), followed by the vHP-SD protocol. In contrast, the HP-SD-15 protocol resulted in the highest values across all three parameters. Lesion depth, maximum lesion diameter, and lesion volume increased significantly with higher contact force (*p* < 0.001, *p* = 0.002, and *p* = 0.003, respectively). However, the absolute changes in these lesion dimensions were relatively small compared to those observed with power-controlled RF catheters, likely due to the effect of temperature-based power regulation.

**Supplementary Information:**

The online version contains supplementary material available at 10.1038/s41598-025-06533-5.

## Introduction

Radiofrequency (RF) ablation remains the cornerstone of catheter ablation procedures in cardiac electrophysiology. Despite numerous technical innovations aimed at achieving the primary goal of ablation—a durable and transmural lesion—this objective is often not fully realized^1,2^. While the mechanisms behind incomplete ablation are not yet fully understood^3^, several contributing factors have been identified, including catheter movement, contact force (CF) leading to inadequate lesion formation^1–3^, reversible myocardial damage^3^, and regional variations in atrial tissue thickness^4–7^.

For this reason, a thorough understanding of the factors influencing lesion formation is crucial for achieving safe, effective, and optimal ablation therapy. It is well established that various parameters, including irrigation^8^, the power level and duration of energy delivery^9,10^, CF^11^, and power modulation of the RF current^8^ during ablation significantly influence lesion metrics.

High-power, short-duration (HP-SD, 50 watts 10–15 s) ablation is a clinically established and widely applied strategy in atrial fibrillation ablation, offering a favorable balance between lesion efficacy, procedural safety, and workflow efficiency. Studies have shown that this protocol yields predictable lesion dimensions, typically producing moderately deep but broad lesions that are well suited for ablation in thin-walled atrial structures^3,12^. Moreover, its application has been associated with low complication rates and high first-pass isolation success in pulmonary vein isolation^13^. As a result, the HP-SD protocol, using 50 watts for 15 s, has become a standard clinical benchmark in many ablation strategies.

Beyond the conventional moderate-power, moderate-duration (MP-MD) and HP-SD RF ablation protocols, the latest advancement in RF ablation is a very high-power, short-duration (vHP-SD; 90 watts, 4 s) protocol. This approach is employed only in combination with a CF-sensing, irrigated-tip, and temperature-controlled ablation catheter^13^. Experimental data on lesion metrics of the vHP-SD protocol remain limited. Ex vivo findings have shown that lesion dimensions created with vHP-SD seems to bei largly unaffected by changes in contact force, indicating minimal CF-dependence under temperature-regulated power control^11^. Additional in vivo studies using a temperature-controlled vHP-SD setting have emphasized the procedural importance of tighter interlesion spacing with vHP-SD, due to its smaller lesion geometry, which may help reduce gap-related reconnection and improve lesion set durability^14,15^. These observations highlight the need for a detailed understanding of lesion geometry using different ablation protocols and RF energy control modes to support protocol-specific optimization strategies.

In that regard, our study was designed to address the biophysical characteristics of individual lesions (depth, width, volume) and their modulation by CF, using the using the latest irrigated, temperature-controlled ablation catheter in a porcine ex vivo model. This approach allows for a detailed, direct comparison of lesion geometry of the HP-SD and vHP-SD ablation protocols and provides practical data on how lesion metrics behave under real-time temperature-regulated power delivery in RF ablation.

## Methods

### Experimental setup

**Fig. 1:**
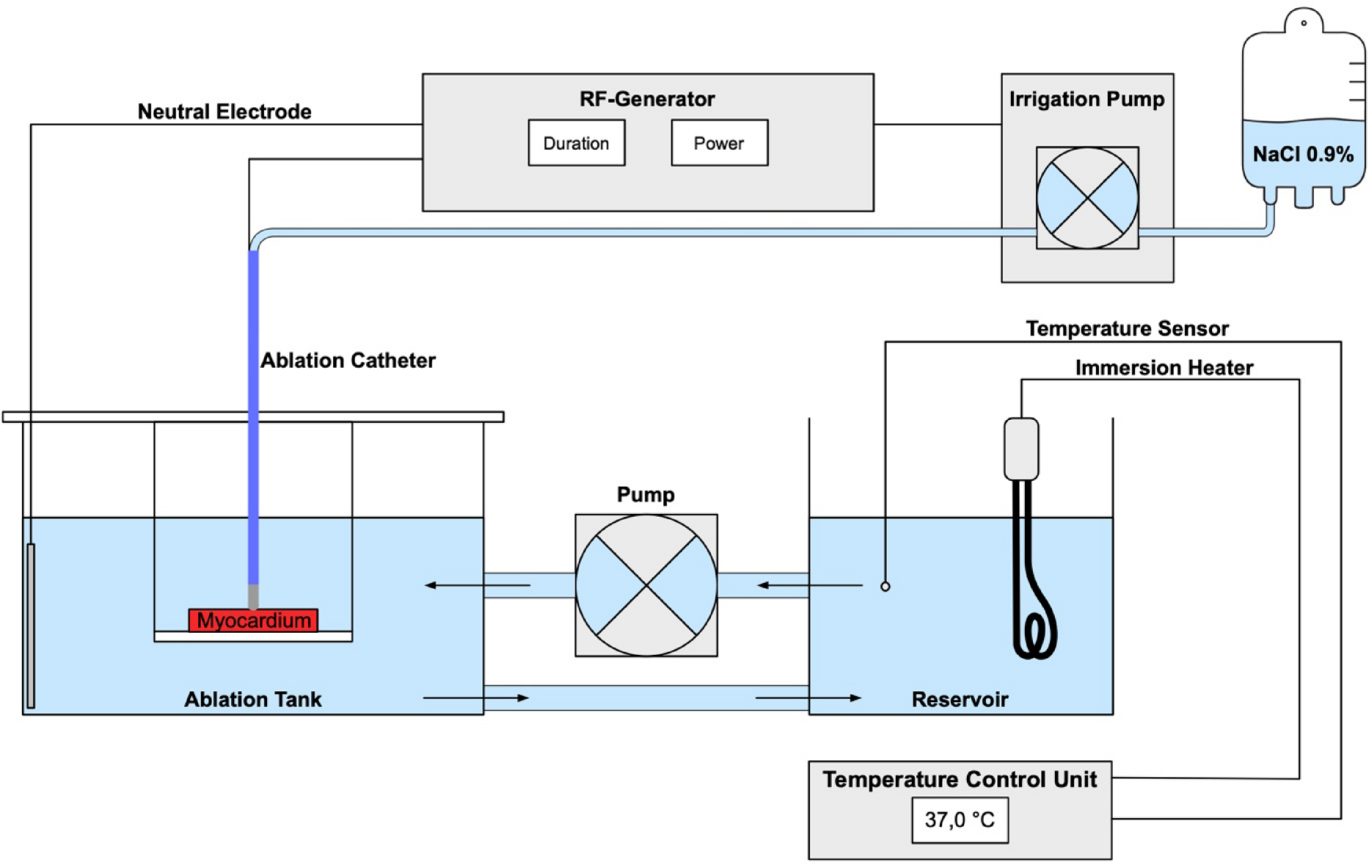
Schematic overview of the experimental set-up.

A schematic overview of the experimental setup is shown in Fig. 1. No live animals were utilized in this study. The experiments were conducted in a saline bath (0.9% NaCl) to simulate the conductive properties of blood. To mimic in vivo blood flow conditions, fluid circulation was maintained using a pump at a flow rate of 5000 mL/min. The bath temperature was continuously regulated at 37.0 °C using a temperature control unit, an immersion heater, and a temperature sensor to ensure physiological conditions during the ablation procedures.

The figure provides a schematic overview of the experimental setup. The main components include an ablation tank and a reservoir tank, both filled with 0.9% NaCl. A pump circulates the fluid between the tanks, directing the inflow of the ablation tank toward the ablation site. A temperature control unit, equipped with a sensor placed at the outflow of the reservoir tank, regulates a heater to ensure the fluid is maintained at 37.0 °C. Ablation was performed using an irrigated radiofrequency catheter with temperature-based power control, in conjunction with a radiofrequency generator and an irrigation pump.

### Ablation protocol

Ablations were performed perpendicularly on freshly excised porcine ventricular myocardium (< 4 h post-excision). The porcine hearts used in this study were sourced as byproducts from a local abattoir. No pigs were specifically euthanized for this study. In total 12 porcine hearts were used. Experiments were conducted with at least three independent biological replicates to ensure reproducibility. A constant CF was maintained at six different levels (5, 10, 15, 20, 25, and 30 g) to assess the impact of CF on lesion formation. Three distinct ablation protocols were applied: HP-SD-4 (50 watts for 4 s), HP-SD-15 (50 watts for 15 s) and vHP-SD (90 watts for 4 s).

For each ablation protocol, 20 lesions were created and analyzed per contact force level, resulting in a total of 120 lesions per ablation protocol.

All ablations were conducted using the QDot Micro temperature-controlled ablation catheter (Johnson & Johnson MedTech, Irvine, CA, USA). This catheter features real-time temperature-controlled RF current modulation and is equipped with six thermocouples for continuous temperature monitoring at the catheter tip. RF energy delivery was controlled using the nGEN ablation generator (Johnson & Johnson MedTech, Irvine, CA, USA) in combination with the CARTO 3 system (Version 7.5). For further detail see Table S1.

### Lesion metrics analysis

**Fig. 2:**
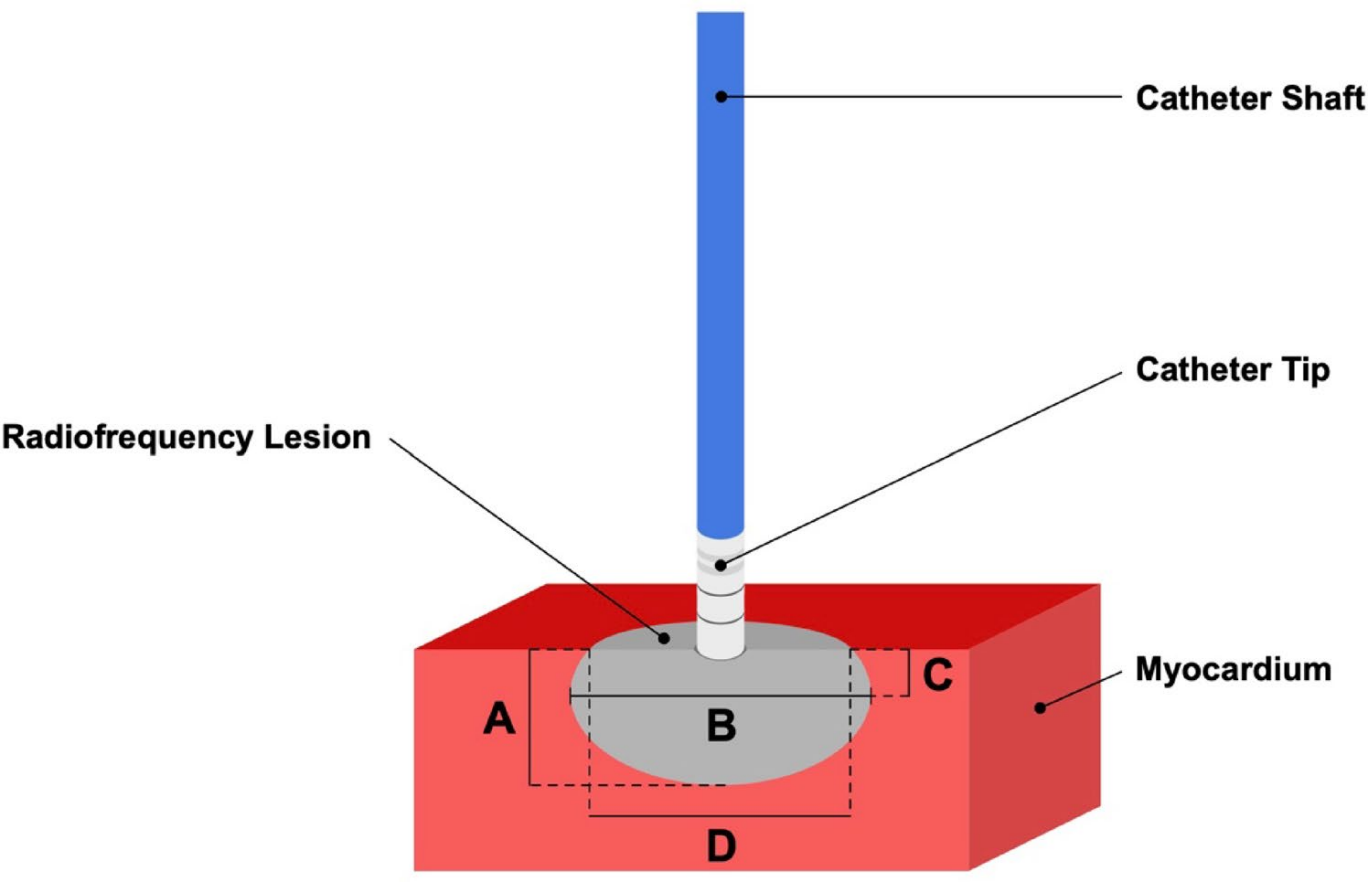
Schematic profile of a radiofrequency lesion. (**A**) Lesion depth was measured from the endocardial surface to the deepest point of the radiofrequency lesion. (**B**) Maximum lesion diameter was measured at the widest point of the lesion. (**C**) Depth to maximum lesion diameter was measured from the endocardial surface to the widest point of the lesion. (**D**) Surface diameter of the radiofrequency lesion.

**Fig. 3:**
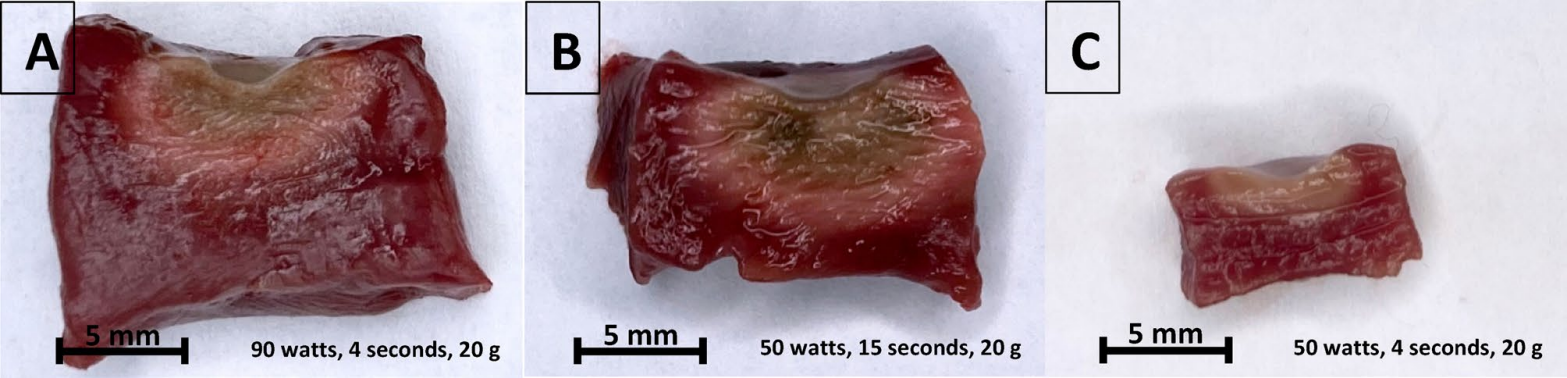
Exemplary images of very high-power, short-duration and a high-power, short-duration radiofrequency lesions on the same scale. Cross-sectional depiction of radiofrequency lesions in porcine ventricular myocardium on the same scale: (**A**) lesion created with 90 watts for 4 s at a contact force of 20 g, (**B**) lesion created with 50 watts for 15 s at a contact force of 20 g, and (**C**) lesion created with 50 watts for 4 s at a contact force of 20 g,

Lesion metrics were assessed macroscopically. First, the myocardium was sectioned at the center of each RF lesion to obtain a cross-sectional view for analysis. Lesion metrics were defined as shown in Fig. 2, which provides a schematic profile of a RF lesion. Figure 3 presents two exemplary images of vHP-SD and HP-SD lesions created in this study.

After cross sectioning the lesions at their maximum surface diameter, images were captured using a digital camera (iPhone SE, 3. Generation, Apple, CA, USA, 12 megapixels) combined with a ring light to ensure optimal illumination. Measurements were conducted using photo-based measurement software (ImageJ, version 1.54 g, National Institutes of Health, USA). To ensure accuracy, the electronic measurement tool was calibrated using a ruler placed at the myocardial level.

The lesion volume (V) was calculated using the following established formula^11^:

$$\:V=\:\left(\frac{1}{6}\right)*\pi\:*\left(a*{b}^{2}+c*\frac{{d}^{2}}{2}\right)$$

### Statistical analysis

Statistical analysis was performed using GraphPad Prism (version 10.2.2, GraphPad Software, Boston, MA, USA) and SAS (version 9.4, SAS Institute, NC, USA). A p-value < 0.05 was considered statistically significant. Normality of numeric variables was assessed using the Shapiro-Wilk test, and data were expressed as mean ± standard deviation (SD). Homogeneity of variances was evaluated using the Levene test.

Inferential statistics was performed using conventional statistical hypothesis testing as well as linear mixed regression modelling. One- and two-way ANOVA along with Student’s t-test for pairwise comparisons of parameters were applied between the ablation protocols, and to assess significant differences within a single ablation protocol across increasing contact force levels. To further validate these results, inference from linear mixed regression analysis was used by means of interpreting the main effects of ablation protocols, CF levels and their interaction. For this, especially linear contrast hypotheses were used.

## Results

### Lesion data

Lesion metrics were compared between the vHP-SD, the HP-SD-4 and the HP-SD-15 protocols across the evaluated contact force range of 5–30 g, with 20 lesions analyzed per contact force level and protocol. When comparing the mean value of all 120 lesions per protocol, the HP-SD-4 protocol resulted in the lowest mean lesion depth, mean maximum lesion diameter, and mean lesion volume, followed by the vHP-SD protocol, while the HP-SD-15 protocol produced the highest values for all three metrics. For further information, see Table 1.

**Table 1:** Overview of mean lesion dimensions across the evaluated contact force range (5–30 g), comparing the very high-power short-duration and high-power short-duration protocols.

Lesion metrics	vHP-SD 90 W/4 s *(n = 120* ^*1*^ *)*	HP-SD-4 50 W/4 s *(n = 120* ^*1*^ *)*	HP-SD-15 50 W/15 s *(n = 120* ^*1*^ *)*	*p*-value vHP-SD vs. HP-SD-4	*p*-value vHP-SD vs. HP-SD-15	*p*-value HP-SD-4 vs. HP-SD-15
**Lesion depth** ^*1*^ *(mean ± SD) [mm]*	3.16 ± 0.41	2.42 ± 0.61	4.49 ± 0.66	< 0.001	< 0.001	< 0.001
**Maximum lesion diameter** ^*1*^ (mean ± SD) [mm]	7.34 ± 0.92	6.02 ± 1.00	9.13 ± 1.59	< 0.001	< 0.001	< 0.001
**Surface diameter** ^*1*^ *(mean ± SD) [mm]*	4.79 ± 0.95	4.08 ± 0.93	4.45 ± 1.02	< 0.001	0.008	0.004
**Lesion volume** ^*1*^ *(mean ± SD) [mm* ^*3*^ *]*	96.4 ± 28.8	52.0 ± 22.5	211.4 ± 95.3	< 0.001	< 0.001	< 0.001
**Mean power output** ^*1*^ *(mean ± SD) [mm* ^*3*^ *]*	59.1 ± 15.6	37.5 ± 3.4	40.5 ± 7.0	< 0.001	< 0.001	< 0.001

*vHP-SD*,* very high-power short-duration*,* HP-SD*,* high-power short-duration; 1: The mean values were calculated based on 120 lesions (= 20 lesions per contact force level (5*,* 10*,* 15*,* 20*,* 25*,* and 30 g)).*

**Fig. 4:**
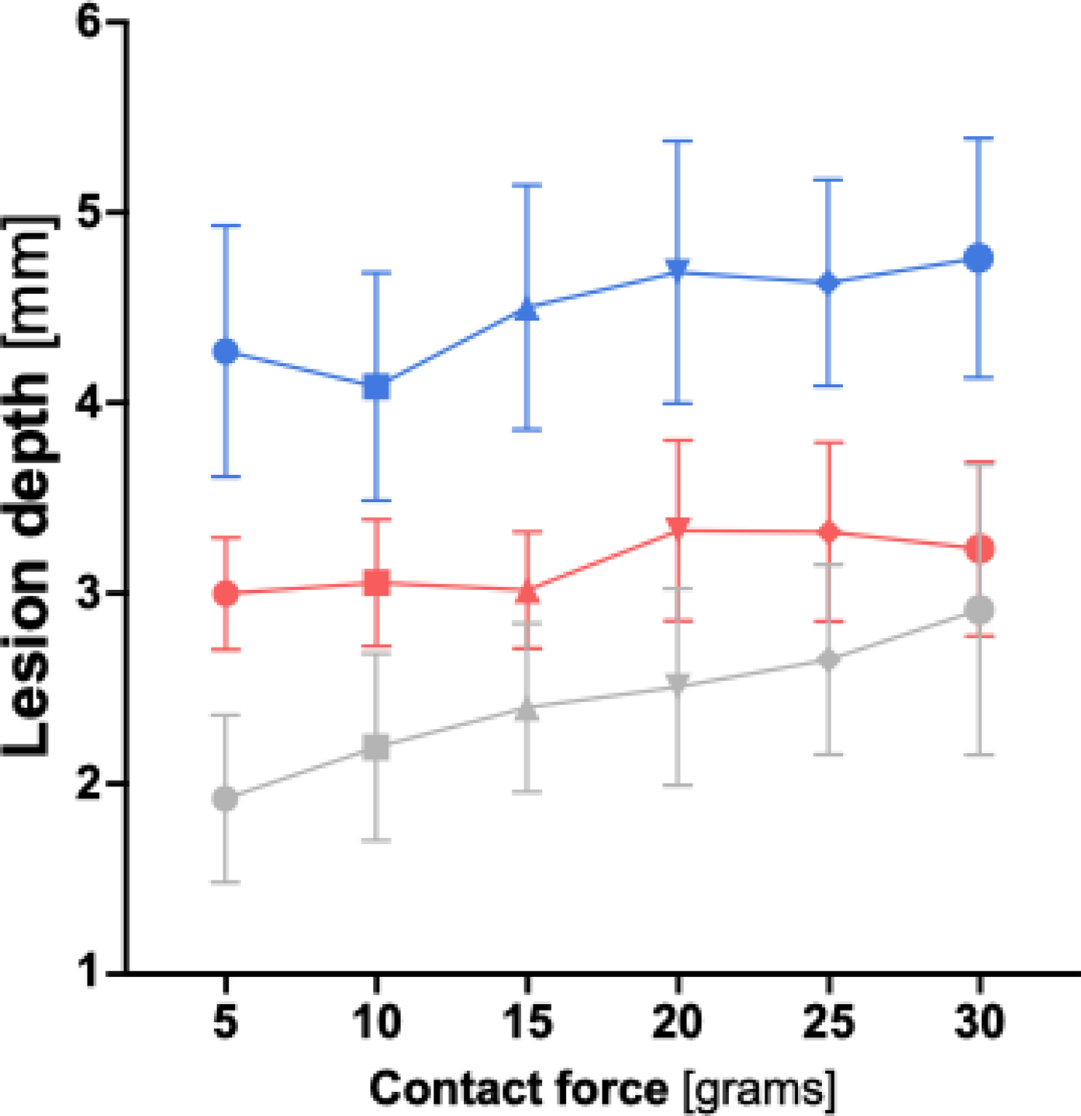
Graphical comparison of lesion depth depending on contact force for the very high-power short-duration (4 s) and the high-power short-duration protocol (4 and 15 s). The diagram illustrates the mean lesion depth for both the very high-power short-duration and high-power short-duration protocols in this study, depending on the applied contact force. The red line represents the data from the very high-power short-duration protocol (90 watts, 4 s), while the blue line corresponds to the high-power short-duration protocol (50 watts, 15 s).

When comparing lesion depth across the three ablation protocols, the HP-SD-4 protocol consistently produced the lowest values, followed by the vHP-SD protocol, with the HP-SD-15 protocol yielding the highest lesion depths at every measured contact force (CF) (see Fig. 4). All three protocols showed a significant increase in lesion depth with increasing CF (*p* < 0.001). For a detailed overview of mean lesion depths according to ablation protocol and across the investigated contact force range, see Table S2 and Table S3 in the supplementary materials.

**Fig. 5:**
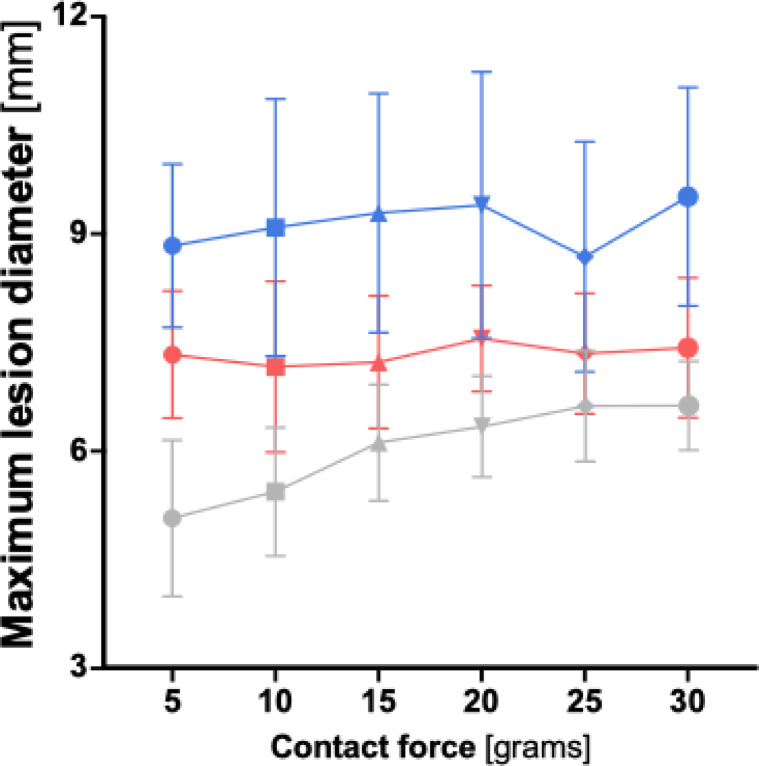
Graphical comparison of maximum lesion diameter depending on contact force for the very high-power short-duration (4 s) and the high-power short-duration protocol (4 and 15 s). The diagram illustrates the mean maximum lesion diameter for both the very high-power short-duration and high-power short-duration protocols in this study, depending on the applied contact force. The red line represents the data from the very high-power short-duration protocol (90 watts, 4 s), while the blue line corresponds to the high-power short-duration protocol (50 watts, 15 s).Graphical comparison of maximum lesion diameter depending on contact force for the very high-power short-duration (4 s) and the high-power short-duration protocol (4 and 15 s).

When comparing maximum lesion diameter across the three ablation protocols, the HP-SD-4 protocol consistently produced the lowest values, followed by the vHP-SD protocol, with the HP-SD-15 protocol yielding the highest maximum lesion diameter at every measured contact force (CF) (see Fig. 5). All three protocols showed a significant increase in maximum lesion diameter with increasing CF (*p* = 0.002). For a detailed overview of the maximum lesion diameter according to the ablation protocol and across the investigated contact force range, see Table S2 and Table S4 in the supplementary materials.

**Fig. 6:**
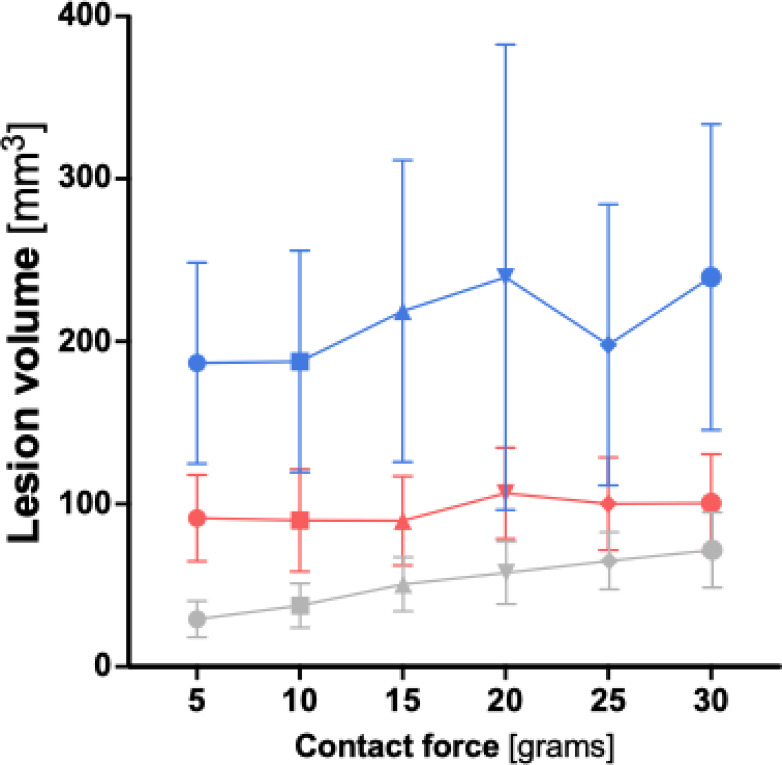
Graphical comparison of the calculated lesion volume depending on contact force for the very high-power short-duration (4 s) and the high-power short-duration protocol (4 and 15 s). The diagram illustrates the mean lesion volume for both the very high-power short-duration and high-power short-duration protocols in this study, depending on the applied contact force. The red line represents the data from the very high-power short-duration protocol (90 watts, 4 s), while the blue line corresponds to the high-power short-duration protocol (50 watts, 15 s).

When comparing lesion volume across the three ablation protocols, the HP-SD-4 protocol consistently produced the lowest values, followed by the vHP-SD protocol, with the HP-SD-15 protocol yielding the highest lesion volume at every measured contact force (CF) (see Fig. 6). All three protocols showed a significant increase in lesion volume with increasing CF (*p* = 0.003). For a detailed overview of the lesion volume according to ablation protocol and across the investigated contact force range, see Table S2 and Table S5 in the supplementary materials.

### Power titration data

**Fig. 7:**
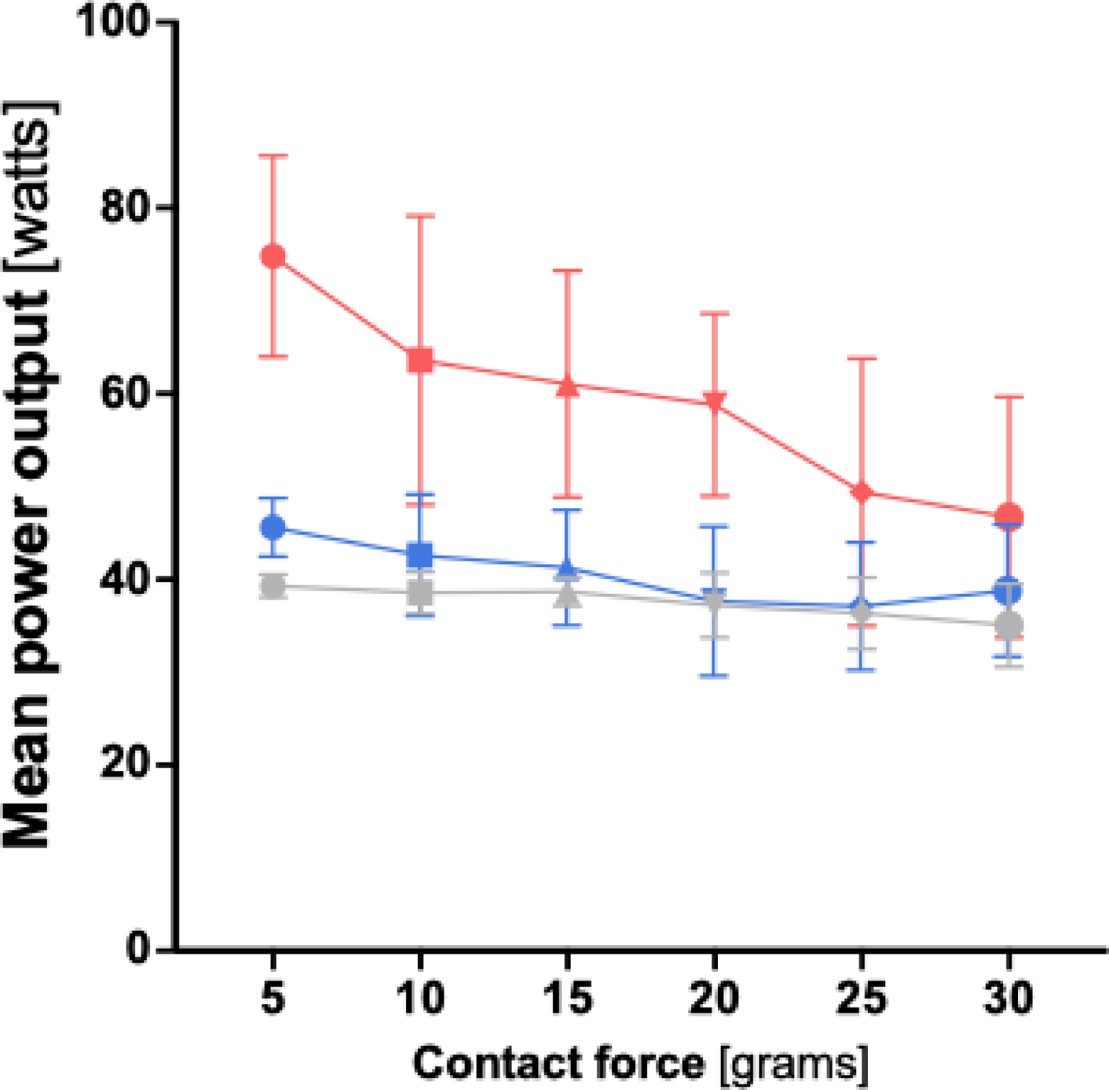
Graphical comparison of the mean power output depending on contact force for the very high-power short-duration (4 s) and the high-power short-duration protocol (4 and 15 s). The diagram illustrates the mean power output for both the very high-power short-duration and high-power short-duration protocols in this study, depending on the applied contact force. The red line represents the data from the very high-power short-duration protocol (90 watts, 4 s), while the blue line corresponds to the high-power short-duration protocol (50 watts, 15 s).

When analyzing the mean energy output of all three ablation protocols, it becomes evident that increasing CF leads to a significant decrease in mean energy output (in all three protocols *p* < 0.001). When comparing mean energy output (measured in watts) across the three ablation protocols, the HP-SD-4 protocol shows the the lowest values, followed by the HP-SD-15 protocol, with the vHP-SD protocol yielding the highest mean energy output at every measured contact force (CF) (see Fig. 7, Table S2, and Table S6).

## Discussion

This study provides a direct comparison of lesion geometry between the vHP-SD and HP-SD ablation protocols using a modern irrigated, temperature-controlled RF catheter in a standardized porcine ex vivo model. Across all measured parameters, lesions created with the vHP-SD protocol were significantly smaller in depth, maximum diameter, and volume compared to those created with the HP-SD-15 protocol. Furthermore, lesion size was only minimally influenced by increasing contact force (CF), regardless of the protocol.

Lesion depth in the vHP-SD protocol was significantly lower in comparion to the HP-SD-15 protocol, despite its higher power delivery, which we attribute to the much shorter RF application time. This observation is consistent with the established principle that lesion depth depends not only on power but also on thermal latency, which is achieved with longer application duration^10,16^. Additional data using 50 watts for 4 s from this study support this interpretation, demonstrating that lesion size remained consistently smaller than with both the HP-SD-15-second and vHP-SD protocols under temperature-controlled RF energy delivery. These findings underscore the central role of RF duration in lesion formation, even when using temperature-controlled RF catheters, and align with prior experimental and in vivo studies showing that shorter-duration, high-power ablation produces shallower lesions than longer-duration protocols^10,12,16^.

Although the vHP-SD protocol limits ablation duration to 4 s to reduce the risk of steam pops, Yamaguchi et al. demonstrated that deeper lesions can still be achieved through repetitive applications using this protocol^17^. While the clinical use of sequential ablations remains exploratory, this approach may offer a strategy to enhance lesion durability in thicker atrial regions, even when using the vHP-SD protocol^17^.

Although previous studies have evaluated lesion metrics using temperature-controlled vHP-SD protocols, comparative CF-dependent data between vHP-SD and HP-SD under standardized conditions remain scarce. For the vHP-SD protocol Yamaguchi et al.^11^ found that lesion dimensions at 90 watts, 4 s were largely unaffected by moderate CF changes in ex vivo experiments using temperature-controlled catheters^11^. In contrast, it is well established that lesion depth in power-controlled RF ablation significantly increases with greater CF, often by several millimeters^16,18^. In that regard, our study supports previous RF ablation findings and further extends current knowledge by directly comparing RF ablation using the vHP-SD and HP-SD-15 protocols under identical conditions. While we observed a significant effect of CF on lesion metrics, this effect remained modest under temperature-controlled RF ablation with the vHP-SD and HP-SD protocols. The limited influence of CF is likely attributable to power downregulation in temperature-controlled settings. This downregulation may result from a rapid rise in catheter-tip temperature due to increased tissue contact at higher CF, which reduces the effectiveness of blood flow-mediated cooling.

When comparing the mean depth of HP-SD lesions created with a temperature-controlled ablation catheter in this study to those reported using power-controlled catheters in other studies, the achievable lesion depths appear largely comparable^12,16^. This suggests that, overall, there is no significant difference in mean lesion depth between temperature-controlled and standard power-controlled RF ablation. This finding aligns with observations from the study group of Takigawa et al.^19^.

In addition to knowledge about the achievable lesion depth, understanding the achievable lesion width is also crucial for successful ablation treatment. Clinical and preclinical studies have demonstrated that interlesion spacing must be narrower, when using low ablation index guided HP-SD or with vHP-SD ablation to maintain lesion continuity. Bortone et al. reported that durable conduction block with both 50 watts at low ablation index (AI ≈ 370) and vHP-SD required spacing < 4 mm^14,15^. Our results offer a mechanistic rationale for these findings: the smaller maximum lesion diameter of vHP-SD and HP-SD-4 lesions in comparison to the HP-SD-15 protocol may result in insufficient overlap at standard interlesion spacing.

Recent clinical trials reinforce the anatomical considerations implied by our findings. In the POWER PLUS trial^20^, vHP-SD was associated with a slightly reduced first-pass isolation rate at anterior and carinal sites, regions known for greater wall thickness. At the same time, the vHP-SD protocol has been shown, both in the POWER PLUS trial and in other studies, to significantly shorten overall procedure duration compared to conventional ablation strategies, primarily due to reduced energy application time and fewer catheter repositioning^13,20–22^. These observations correspond to our results, which suggest that vHP-SD may be less suited for thick myocardial targets unless sequential application or tighter spacing is employed, while still offering procedural efficiency in anatomically favorable regions.

Interestingly, within the range of CF investigated, lesion width in the HP-SD and vHP-SD protocols were only marginally affected, supporting the concept that temperature-controlled systems provide more consistent lesion metrics across varying contact force conditions. This effect was also observed by Yamaguchi et al.^17^.

Together, our data indicate that vHP-SD is better suited for thin-walled areas such as the posterior or roof of the left atrium, where shallow, wide lesions can provide effective isolation while minimizing the risk of collateral injury, like esophageal injury or atrioesophageal fistula^4^. In contrast, HP-SD-15 appears to be better suited for anterior wall regions and likely for redo ablation procedures, where deeper lesions are required to achieve transmurality.

## Limitations

This study was conducted using an ex vivo porcine ablation model, which, while allowing precise control of ablation parameters, does not fully replicate the physiological conditions encountered in vivo. To minimize tissue degradation, freshly excised (< 4 h) porcine myocardium was used; however, interspecies differences in myocardial thickness and conductivity limit direct extrapolation to the human atrium^9^.

The absence of active myocardial perfusion eliminates the dynamic cooling effect of intramural blood flow, potentially leading to overestimation of lesion depth and size. Although a circulating temperature-controlled saline bath was used to mimic blood flow, this can only partially simulate in vivo convective cooling.

Furthermore, the model does not account for respiratory motion, cardiac contraction, or catheter instability, all of which can influence lesion formation, particularly with short-duration protocols^11,23^. The flat myocardial geometry used also differs from the curved, trabeculated atrial anatomy seen clinically, which may affect energy distribution and lesion overlap.

Despite these limitations, the ex vivo model enabled systematic, force-dependent comparison of lesion geometry under standardized conditions, offering mechanistic insights that support interpretation of clinical and in vivo findings.

## Conclusion

This study demonstrates that RF lesions created with the vHP-SD protocol are smaller in depth, width, and volume compared to those produced by the HP-SD-15 protocol when using a temperature-controlled ablation catheter. These findings suggest that the vHP-SD protocol is better suited for ablating thinner myocardial regions, where precise lesion depth is essential, while the HP-SD-15 protocol is more appropriate for achieving deeper atrial lesions.

Additionally, lesion characteristics, whether using the vHP-SD or HP-SD protocol, are only minimally influenced by CF when a temperature-controlled RF ablation catheter is used.

Recognizing these distinctions can help refine ablation strategies for specific cardiac regions, enhancing both procedural safety and efficacy.

## Electronic supplementary material

Below is the link to the electronic supplementary material.

Supplementary Material 1

## Data Availability

The data presented in this study are available on request from the corresponding author.
