# Supplementary material for: Comparative lesion metrics analysis of very high power and high power short duration radiofrequency ablation in a Porcine ex vivo model
Source: Sci Rep. 2025 Jun 20;15:20215. doi: 10.1038/s41598-025-06533-5 (PMC12181290; doi:10.1038/s41598-025-06533-5)
Supplement: Supplementary file 1 — Supplementary Material 1 [file 41598_2025_6533_MOESM1_ESM.docx]

**SUPPLEMENT - Comparative lesion metrics analysis of very high power and high power short duration radiofrequency ablation in a porcine ex vivo model**

**Table S1:** Overview over the ablation data

|  | vHPSD Protocol | HP-SD Protocol |
| --- | --- | --- |
| Used Catheter | QDot Micro | QDot Micro |
| Power Control Mode | Temperature controlled | Temperature controlled |
| Power | 90 watts | 50 watts |
| Duration | 4 seconds | 15 or 4 seconds |
| Initial irrigation flow | 2 ml/minute | 2 ml/minute |
| Ablation irrigation flow | 8 ml/minute | 4 – 15^1^ ml/minute |
| Catheter contact force | 5, 10, 15, 20, 25, 30 grams | 5, 10, 15, 20, 25, 30 grams |
| Catheter orientation | perpendicular | perpendicular |

*1: High flow minimum and high flow maximum*

**Table S2:** Statistical comparison (linear mixed regression analysis) of lesion metrics using a temperature-controlled radiofrequency catheter between the very high-power short-duration (4 seconds) and the high-power short-duration (4 and 15 seconds) protocol, depending on contact force.

| **Lesion depth** | **5 g** | **10 g** | **15 g** | **20 g** | **25 g** | **30 g** |
| --- | --- | --- | --- | --- | --- | --- |
| **HP-SD-15 vs. HP-SD-4** | < 0.001 | < 0.001 | < 0.001 | < 0.001 | < 0.001 | < 0.001 |
| **HP-SD-15 vs. vHP-SD** | < 0.001 | < 0.001 | < 0.001 | < 0.001 | < 0.001 | < 0.001 |
| **HP-SD-4 vs. vHP-SD** | < 0.001 | < 0.001 | < 0.001 | < 0.001 | < 0.001 | 0.058 |
|  | | | | | | |
| **Maximum lesion diameter** | **5 g** | **10 g** | **15 g** | **20 g** | **25 g** | **30 g** |
| **HP-SD-15 vs. HP-SD-4** | < 0.001 | < 0.001 | < 0.001 | < 0.001 | < 0.001 | < 0.001 |
| **HP-SD-15 vs. vHP-SD** | < 0.001 | < 0.001 | < 0.001 | < 0.001 | < 0.001 | < 0.001 |
| **HP-SD-4 vs. vHP-SD** | < 0.001 | < 0.001 | 0.003 | 0.001 | 0.047 | 0.028 |
|  | | | | | | |
| **Lesion volume** | **5 g** | **10 g** | **15 g** | **20 g** | **25 g** | **30 g** |
| **HP-SD-15 vs. HP-SD-4** | < 0.001 | < 0.001 | < 0.001 | < 0.001 | < 0.001 | < 0.001 |
| **HP-SD-15 vs. vHP-SD** | < 0.001 | < 0.001 | < 0.001 | < 0.001 | < 0.001 | < 0.001 |
| **HP-SD-4 vs. vHP-SD** | < 0.001 | 0.005 | 0.034 | 0.008 | 0.056 | 0.116 |
|  | | | | | | |
| **Mean power output** | **5 g** | **10 g** | **15 g** | **20 g** | **25 g** | **30 g** |
| **HP-SD-15 vs. HP-SD-4** | 0.019 | 0.1313 | 0.342 | 0.867 | 0.780 | 0.162 |
| **HP-SD-15 vs. vHP-SD** | < 0.001 | < 0.001 | < 0.001 | < 0.001 | < 0.001 | 0.003 |
| **HP-SD-4 vs. vHP-SD** | < 0.001 | 0.005 | 0.034 | 0.008 | 0.056 | 0.116 |

**Table S3:** Overview of lesion depth by ablation protocol and contact force.

| **Lesion depth** | **vHP-SD**  90 W / 4 s | **HP-SD-4**  50 W / 4 s | **HP-SD-15**  50 W / 15 s |
| --- | --- | --- | --- |
| **5g** | 0.300 ± 0.029 mm | 0.192 ± 0.044 mm | 0.427 ± 0.066 mm |
| **10g** | 0.306 ± 0.033 mm | 0.219 ± 0.049 mm | 0.409 ± 0.060 mm |
| **15g** | 0.302 ± 0.031 mm | 0.240 ± 0.044 mm | 0.450 ± 0.064 mm |
| **20g** | 0.333 ± 0.047 mm | 0.251 ± 0.052 mm | 0.469 ± 0.069 mm |
| **25g** | 0.332 ± 0.047 mm | 0.265 ± 0.050 mm | 0.463 ± 0.054 mm |
| **30g** | 0.323 ± 0.046 mm | 0.292 ± 0.076 mm | 0.476 ± 0.063 mm |

**Table S4:** Overview of maximum lesion diameter by ablation protocol and contact force.

| **Maximum lesion diameter** | **vHP-SD**  90 W / 4 s | **HP-SD-4**  50 W / 4 s | **HP-SD-15**  50 W / 15 s |
| --- | --- | --- | --- |
| **5g** | 0.733 ± 0.088 | 0.506 ± 0.108 | 0.883 ± 0.113 |
| **10g** | 0.716 ± 0.118 | 0.543 ± 0.089 | 0.908 ± 0.178 |
| **15g** | 0.723 ± 0.092 | 0.610 ± 0.080 | 0.928 ± 0.165 |
| **20g** | 0.755 ± 0.073 | 0.633 ± 0.070 | 0.939 ± 0.184 |
| **25g** | 0.735 ± 0.083 | 0.661 ± 0.077 | 0.868 ± 0.159 |
| **30g** | 0.743 ± 0.097 | 0.661 ± 0.061 | 0.951 ± 0.151 |

**Table S5:** Overview of lesion volume by ablation protocol and contact force.

| **Lesion volume** | **vHP-SD**  90 W / 4 s | **HP-SD-4**  50 W / 4 s | **HP-SD-15**  50 W / 15 s |
| --- | --- | --- | --- |
| **5g** | 91 ± 27 mm^3^ | 29 ± 11 mm^3^ | 186 ± 62 mm^3^ |
| **10g** | 90 ± 31 mm^3^ | 38 ± 14 mm^3^ | 187 ± 68 mm^3^ |
| **15g** | 90 ± 27 mm^3^ | 51 ± 17 mm^3^ | 218 ± 93 mm^3^ |
| **20g** | 107 ± 28 mm^3^ | 58 ± 19 mm^3^ | 239 ± 143 mm^3^ |
| **25g** | 100 ± 29 mm^3^ | 65 ± 18 mm^3^ | 198 ± 86 mm^3^ |
| **30g** | 101 ± 30 mm^3^ | 72 ± 23 mm^3^ | 239 ± 94 mm^3^ |

**Table S6:** Overview of mean power output by ablation protocol and contact force.

| **Mean power output** | **vHP-SD**  90 W / 4 s | **HP-SD-4**  50 W / 4 s | **HP-SD-15**  50 W / 15 s |
| --- | --- | --- | --- |
| **5g** | 75 ± 11 watts | 39 ± 1 watts | 46 ± 3 watts |
| **10g** | 64 ± 16 watts | 39 ± 2 watts | 43 ± 7 watts |
| **15g** | 61 ± 12 watts | 39 ± 2 watts | 41 ± 6 watts |
| **20g** | 59 ± 10 watts | 37 ± 3 watts | 38 ± 8 watts |
| **25g** | 49 ± 14 watts | 36 ± 4 watts | 37 ± 7 watts |
| **30g** | 47 ± 13 watts | 35 ± 4 watts | 39 ± 7 watts |
